# Store-Operated Calcium Entry As an Important Mechanism of Tumor Adaptation to an Aggressive Microenvironment

**DOI:** 10.32607/actanaturae.27574

**Published:** 2025

**Authors:** A. Yu. Skopin, E. V. Kaznacheyeva

**Affiliations:** Institute of Cytology, Russian Academy of Sciences, St. Petersburg, 194064 Russia

**Keywords:** calcium, store-operated calcium entry, STIM, Orai, malignant transformation, tumor microenvironment, calcium signaling

## Abstract

Calcium signaling ensures efficient cellular functioning; calcium homeostasis disruption leaves behind detrimental sequelae for the cell both under calcium excess and deficiency conditions. Malignant transformation is accompanied by significant alterations in the expression of the proteins critical for store-operated calcium entry, resulting in the dysregulation of calcium signaling. It is plausible that a remodeling of intracellular signal transduction pathways in cancer cells is required in order to accelerate metabolic processes, as well as fuel further tumor growth and invasion. Meanwhile, fine-tuning of calcium signaling is observed under both normal and pathological conditions. In this context, research into the changes accompanying signal transduction within the tumor microenvironment is a key aspect of the investigation of the role of calcium signaling in tumor development. Factors characteristic of the tumor microenvironment were shown to have a significant effect on the function of calcium channels and the proteins that regulate calcium signaling. Major, adverse microenvironmental factors, such as acidification, elevated levels of reactive oxygen species and hypoxia, have a bearing on the store-operated calcium entry. It is crucial to understand whether changes in the expression of the key SOCE components represent an adaptation to the microenvironment or a result of carcinogenesis.

## INTRODUCTION

The tumor microenvironment is shaped by various cell types, both cancer and non-cancer ones (e.g., immune cells). Carcinogenesis is under the continuous influence of neighboring cells, soluble factors, and the extracellular matrix. The soluble factors include nutrients, oxygen, reactive oxygen (ROS) and nitrogen species, ATP, cytokines, growth factors, chemokines, various ions (e.g., Ca^2+^ and H^+^), metabolic waste products of cancer cells, etc. [1, 2]. Changes in the intracellular calcium concentration affect proliferation, apoptosis, energy metabolism, and the invasiveness of cancer cells, thereby playing a pivotal role in tumor growth and development [3, 4, 5]. To date, a significant amount of data has accumulated indicating the presence of alterations in calcium signaling during tumor transformation. The expression levels of the proteins involved in calcium signaling are known to change during the development of pathological processes. Meanwhile, it remains unclear whether these changes in calcium signaling are driven by adaptation to the tumor microenvironment, where signaling plays a pivotal role, or by changes in the expression levels of specific proteins involved in calcium signal transduction. It seems that both mechanisms − and probably combinations of the two − need consideration.

**Store-operated calcium entry**

**Fig. 1:**
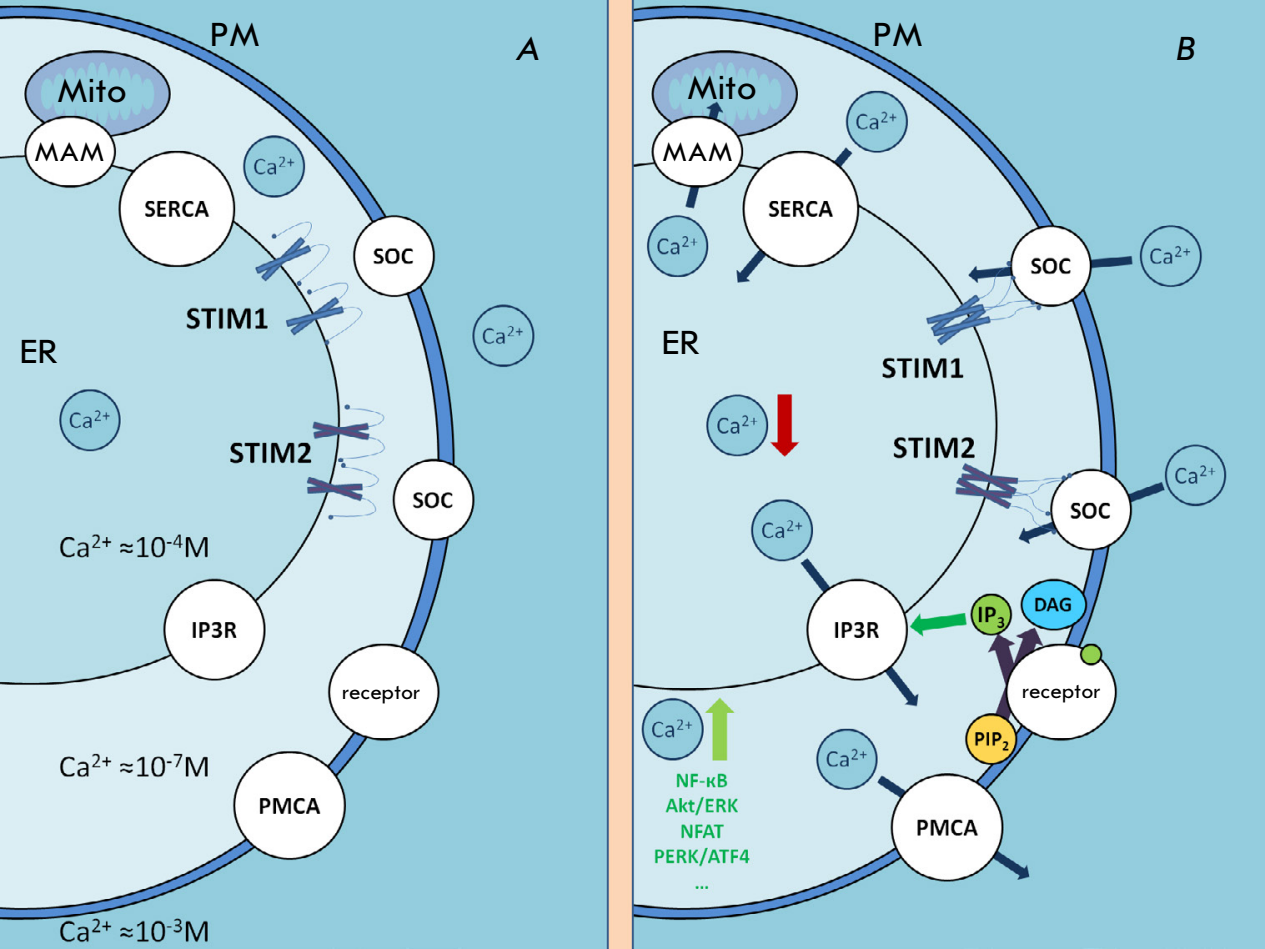
The schematic of store-operated calcium entry. (*A*) The region of close contact between the plasma membrane and the ER membrane at rest. (*B*) Activation of plasma membrane (PM) receptors stimulates IP3 production and calcium release from the ER via the IP3 receptor. A drop in the calcium concentration within the store causes clustering and conformational changes in STIM proteins, as well as the activation of SOC. Calcium entering the cell can activate signaling pathways, refill the ER calcium store by SERCA pumps, and supply mitochondria (Mito) through membrane contact sites (MAMs). Excess calcium is extruded from the cells primarily by PMCAs

Store-operated calcium channels (SOC) residing in the plasma membrane are among the major pathways of calcium entry into non-excitable cells and are widely expressed in various cell types (*Fig. 1*) [6]. SOC activity is vital for the replenishment of calcium stores in the endoplasmic reticulum (ER) and for the transduction of a multitude of intracellular signals [7]. The entry of extracellular calcium into the cell in response to the depletion of intracellular calcium stores is termed store-operated calcium entry (SOCE). STIM and Orai proteins [8], as well as certain members belonging to the TRPC family, are the key molecular components of SOCE [9, 10, 11]. Orai and TRPC form calcium channels in the plasma membrane, while STIM proteins are primarily ER-localized proteins with a single transmembrane domain which function as sensors of the calcium concentration in the ER [12]. A decline in the calcium concentration in the ER is followed by conformational changes, oligomerization, and the clustering of STIM proteins. They were shown to translocate to the area of close contact between the ER membrane and the plasma membrane, where they interact with SOC channels and activate them, thereby mediating the store-operated calcium entry [13].

Two homologs of the STIM protein are expressed in humans: STIM1 and STIM2. Both predominantly reside in the ER membrane, although a small amount of STIM1 is found on the plasma membrane. Both STIM proteins have a similar structure: composed of an N-terminal calcium-binding domain within the ER lumen, a single transmembrane segment, and a C-terminal cytoplasmic domain responsible for protein– protein interactions [14]. In vertebrates, STIM1 and STIM2 are expressed in all cell types; they function as sensors of the endoplasmic reticulum luminal calcium and activators of SOC. Unlike STIM1, STIM2 is confined exclusively to the ER membrane. STIM2 is known to be a weaker activator of Orai1 than STIM1 but a more sensitive Ca^2+^ sensor in the ER lumen. The dissociation constant of STIM2 for Ca^2+^ (500–800 μM) is significantly higher than that of STIM1 (200–00 μM) [15]. It is believed that the primary physiological role of STIM2 is to stabilize basal calcium levels in the cytosol and ER [16]. Furthermore, the STIM2 protein mediates various store-dependent and store-independent SOC activation mechanisms and can inhibit SOCE through alternative splicing products [17, 18].

SOCE possess a broad range of regulatory mechanisms. SOC in the plasma membrane is characterized by a set of electrophysiological properties, regulatory mechanisms, and susceptibility to factors such as acidification, hypoxia, and reactive oxygen species. These channels are activated by STIM proteins that differ in their calcium sensitivity and ability to activate Orai channels [19]. Furthermore, SOC can be categorized into groups activated either by STIM1 or STIM2 [20]. Another level of regulation, which is still poorly understood, involves various adapter proteins and lipids residing at the contact sites between the plasma membrane and the endoplasmic reticulum (e.g., cholesterol, IP3 receptor, adapter proteins of the Homer family, or cytoskeletal proteins) [14, 21, 22, 23].

Importantly, basal calcium concentrations in the cytosol and the ER stores primarily depend on SOCE and significantly affect overall cellular calcium signaling.

Hence, there are several levels of regulation and a broad range of possibilities for fine-tuning the SOCE mechanism to specific conditions. A limited number of reports on alterations in the details of the SOCE mechanism during malignant transformation are available.

**The molecular composition of the mechanism of storage-operated calcium entry in breast cancer**

Multiple publications have demonstrated that the expression profile of proteins, as key SOCE components, is altered in cancer (in particular, breast cancer (BC) (*Table 1*), as well as in colon [24], prostate [25], gastric [25], cervical [27], and oral [28] cancers).

**Table 1:** SOCE gene expression in breast cancer cell lines and control cells

Cell line	MCF-10A	MCF-7	MDA-MB-231	MDA-MB-468	BT-20	BT-474
Characterization of cells	fibrocystic mastopathy	HER2-ER+	TNBC	TNBC	TNBC	HER2+ ER+
Results of functional studies [29, 30, 31]		Orai3 ↑	Orai1 ↑ STIM1 ↑			
Amount of protein normalized with respect to MCF10A [32]	Orai1 Orai2	Orai1↑ Orai2	Orai1↑ Orai2	Orai1 Orai2	Orai1 Orai2↑	
Amount of protein normalized with respect to MCF10A [33]	Orai3 STIM2 TRPC6	Orai3 ↑ STIM2 TRPC6 ↑	Orai3 STIM2 ↓ TRPC6 ↑	Orai3 ↑ STIM2 TRPC6 ↑	Orai3 ↑ STIM2 TRPC6	
Amount of protein [34]		STIM1 STIM2	STIM1 ↑ STIM2 ↓			STIM1 ↓ STIM2 ↑
Amount of mRNA [29]		Orai1 ↑ Orai2 Orai3 ↓	Orai1 ↑ Orai2 Orai3 ↓			
Amount of protein normalized with respect to MCF10A [30]	STIM1 Orai1 Orai3	STIM1 ↓ Orai1 ↓ Orai3 ↑	STIM1 Orai1 Orai3 ↓		STIM1 ↑ Orai1 Orai3 ↓	STIM1↓ Orai1 ↓ Orai3 ↑
Gene expression [35]	TRPC1 ↑	TRPC1	TRPC1 ↑	TRPC1 ↓	TRPC1 ↑	TRPC1 ↓

↑ – upregulated expression;

↓ – downregulated expression.

HER2 – HER2/neu receptor;

ER – endorphin receptor;

TNBC – triple negative breast cancer.

*Table 1* lists the data on the expression of the STIM, Orai, and TRPC proteins in the best studied breast cancer cell lines.

**Fig. 2:**
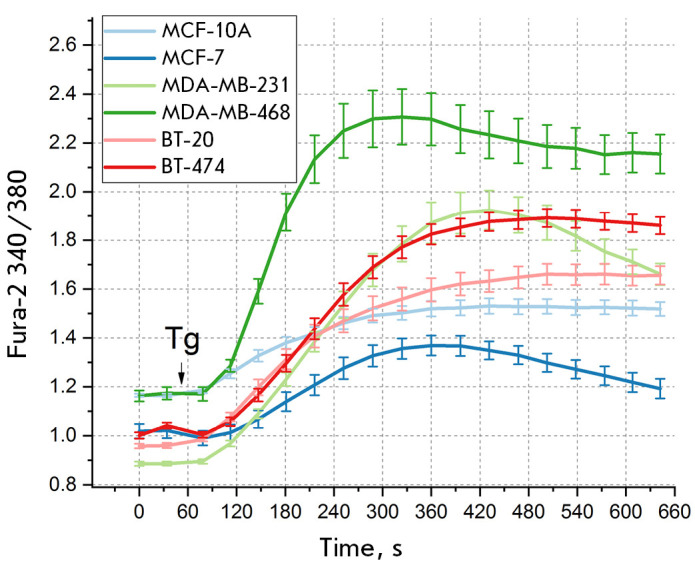
The Tg-induced responses of different breast cancer cell lines in a medium containing 2 mM Ca^2+^. An arrow indicates the time instant of administering 1 μM Tg. The ratio between Fura-2 fluorescence (340 and 380 nm), the mean value, and SEM (*n* = 9–2) are shown

**Fig. 3:**
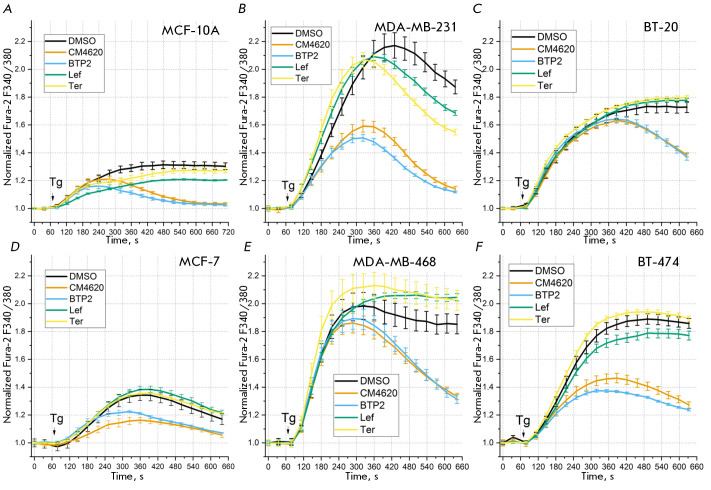
Measurements of the amplitude of the Tg-induced response in the medium containing 2 mM Ca^2+^ after 25-min incubation in the presence of 0.5% DMSO (control), 5 μM CM4620 and BTP2, 50 μM leflunomide (Lef) and teriflunomide (Ter) in cell lines: (*A*) MCF-10A; (*B*) MDA-MB-231; (*C*) BT-20; (*D*) MCF-7; (*E*) MDA-MB-468, and (*F*) BT-474. An arrow indicates the time instant of administering 1 μM Tg. The ratio between Fura-2 fluorescence (340 and 380 nm) normalized with respect to the basal level, the mean value, and SEM (*n *= 9–2) as a function of time are shown. Adapted from ref. [36]

The data summarized in *Table 1* attest to significant variations in the protein composition of the store-operated calcium entry (SOCE) across different breast cancer cell lines. Furthermore, the differences in the expression of the key SOCE components result in changes in the functional characteristics of calcium entry in each particular cell line. We define the calcium response amplitude as the maximum change in the intracellular calcium concentration with respect to basal levels. Studies, including our own research, demonstrate that breast cancer lines are characterized by different calcium response amplitudes and basal calcium concentrations (*Fig. 2*), varying sensitivity to specific (CM4620 and BTP2) and non-specific (leflunomide and teriflunomide) SOCE modulators (*Fig. 3*), as well as microenvironmental conditions (ref. [36] and unpublished data).

Currently, there is no clear understanding of whether these functional changes cause the pathology or are a result of SOCE adaptation to new microenvironmental conditions. Both of these scenarios might be possible. For instance, if a cell becomes able to pump slightly more calcium into the cytosol upon initiation of malignant transformation, it promotes active proliferation and invasion. Alternatively, adjustment of a calcium response in cells within a tumor that has already been formed would lead to the accumulation of cells maximally adapted to these specific conditions.

**Physiological functions of store-operated calcium entry upon malignant transformation**

Numerous examples demonstrate that the key SOCE proteins are involved in the regulation of proliferation, migration, invasion, the epithelial–mesenchymal transition (EMT), neoangiogenesis, and the metastatic spread of cancer cells [37, 38, 39]. Importantly, alterations in protein levels do not trigger a specific transformation pathway common to all cell types. Rather, we observe a degree of disruption of calcium signaling that percolates to various cellular signaling pathways (e.g., the Akt/ERK, NFAT, and PERK/ATF4 pathways), leading to malignant transformation in a manner unique to each tumor type.

In particular, STIM1-mediated calcium entry regulates tumor angiogenesis in Epstein–Barr virus-associated nasopharyngeal carcinoma. The viral-encoded membrane protein LMP1 promotes proliferation, migration, and tubulogenesis by engaging the Akt/ERK pathway. Suppression of STIM1 activity reduces the LMP1 content in exosomes and slows tumor-induced vascular network formation [40]. STIM1 knockout in MDA-MB-231 and other breast cancer cell lines, regardless of their metastatic potential, enhances cell migration, while simultaneously downregulating NFAT1 expression [41]. Orai3 knockout was shown to alter the expression of numerous genes affecting migration and inflammatory/immune responses, including hypoxia-induced ones: ID1, TREM-1, and PGF [42]. In colorectal cancer, downregulated STIM2 expression activates the c-Myc and PERK/ATF4 signaling pathways, thus increasing tumor size and promoting invasion and metastatic spread [43]. SOCE was also shown to be implicated in cell cycle disruption. The Orai3-STIM2 complex ensures successful mitosis in prostate cancer cells, preventing mitotic catastrophe. Suppression of Orai3 expression increases SOCE and causes G2/M phase cell cycle arrest, leading to the activation of the Bax/Bcl-2-mediated apoptotic pathway [44]. The Orai1 protein is overexpressed in patients with B-cell chronic lymphocytic leukemia, compared to normal B cells, contributing to the elevation of basal Ca^2+^ levels through a constitutive activity of SOC. Selective SOCE inhibitors (GSK-7975A and Synta66) block Ca^2+^ entry into cells, inducing apoptosis. Furthermore, Orai1 inhibitors exert an additive/ synergistic effect when used in combination with therapeutics for B-cell chronic lymphocytic leukemia [45]. In SKBR3 and BT20 breast cancer cell lines characterized by upregulated Orai2 expression, this channel modulates NFAT1 and NFAT4 activation in response to agonists. Orai2 knockdown induces the G0/G1 phase cell cycle arrest and reduces the resistance of cells to apoptosis in patients treated with cisplatin [32].

SOCE-forming proteins have also been found to affect the expression of enzymes that regulate oncogenesis. Tumor samples from patients with oral cancer were characterized by upregulated Orai1 expression and, consequently, an increased rate of Ca^2+^ entry into these cells. mRNA analysis revealed that Orai1 regulates many genes encoding oral cancer markers, including metalloproteinases regulated by NFAT4 [46].

Furthermore, there are carcinogenic mechanisms that boost the activity of the SOCE machinery. For instance, upregulated expression of the EHD2 and CAV1/2 proteins is observed in various subtypes of breast cancer. These proteins possibly stabilize plasma membrane caveolae and ensure high cell-surface expression of Orai1, thus leading to increased SOCE that stimulates oncogenesis [47]. In prostate cancer patients, the TSPAN18 protein protects STIM1 against TRIM32-mediated ubiquitination; consequently, STIM1-mediated calcium entry increases, thus intensifying the metastatic spread [48]. Transcriptome analysis data have indicated that *NSUN2 *expression is significantly upregulated in gastric cancer patients. The *NSUN2 *gene regulates the stability of Orai2 mRNA through the 5-methylcytosine modification, thereby promoting Orai2 expression and further development of peritoneal metastasis in gastric cancer patients [49].

This partial list showing the involvement of SOCE in malignant transformation underscores the critical importance of selecting the proper therapeutic target. Along with impairment of SOCE protein expression, there are certain malignant transformations that directly affect the function of SOCE without altering the expression levels of these proteins. Therefore, effective treatment requires agents that can precisely target the function of specific SOCE proteins in a particular situation. For example, while suppression of STIM1 protein activity is beneficial in nasopharyngeal carcinoma, this approach is forbidden in certain types of breast cancer. Reduction of Orai2 activity is a justified strategy for treating specific breast cancer subtypes, as well as gastric cancer.

**The interplay between store-operated calcium entry and mitochondria**

The regulation of mitochondrial activity in cancer cells is one of the fundamental functions of calcium, which is critical for carcinogenesis. The ER is the primary source of calcium for mitochondria, and the structural relationship between these organelles is modulated by various proteins, including calcium channels [50].

By stimulating Ca^2+^-dependent dehydrogenases of the tricarboxylic acid (TCA) cycle, Ca^2+^ enhances ATP production and stabilizes the mitochondrial membrane potential [51]. However, a critical increase in Ca^2+^ concentration is accompanied by an abrupt rise in the permeability of the inner mitochondrial membrane due to the opening of non-selective pores [50], resulting in the disruption of the respiratory chain, ATP hydrolysis, and osmotic swelling, eventually causing the release of apoptogenic molecules and cell death [52].

Hence, ATP production, biosynthesis of phospholipids and steroid hormones, calcium signal transduction, and oxidation of various metabolites in cancer cells all depend on mitochondrial activity, which is regulated, among other factors, by calcium. Upon carcinogenesis, the amount of calcium entering the cell depends on both internal factors (e.g., the expression of the genes encoding the SOCE proteins) and tumor microenvironment factors. If the amount of incoming calcium is insufficient, cancer cells will not receive the energy required to ensure their viability. Conversely, an excessive influx of calcium will lead to the death of cancer cells. This compels the cell to regulate the calcium influx in a constantly changing microenvironment. Next, we will examine how SOCE is affected by tumor microenvironment factors such as reactive oxygen species, acidification, and hypoxia.

## THE EFFECT OF REACTIVE OXYGEN SPECIES ON STORE-OPERATED CALCIUM ENTRY

Reactive oxygen species are a group of molecules formed via partial reduction of O_2_ and that exhibit high reactivity [53]. Mitochondria generating ROS during ATP synthesis are their intracellular source [54]. Thus, elevated ROS levels were observed in triple- negative breast cancer cells, made possible by mitochondrial activity; ROS have been shown to be important for the survival of these cells, since treatment with antioxidants induced their death [55].

ROS have long been considered harmful to cells, believed to cause oxidative damage to various molecules such as proteins, lipids, and DNA. However, we know that moderate ROS levels are essential for physiological cellular functions, including intracellular signaling, proliferation, and immune responses [56]. The cell employs a number of defense mechanisms to strike a balance between intracellular ROS production and elimination [57].

A large number of ROS sources have been found within tumors and their microenvironment. It has been demonstrated that cancer cells can induce a pathological elevation of ROS levels [58]. Oncogene activation, loss of tumor suppressor genes, hypoxia, as well as mitochondrial DNA mutations, can increase the ROS levels in cancer cells [59]. The tumor microenvironment comprises various types of cells recruited upon tumor formation: neutrophils, T cells, macrophages, and fibroblasts. Exposure to cytokines such as interferon-γ (IFNγ), tumor necrosis factor-α (TNFα), and interleukin-1 (IL-1) was shown to enhance ROS production by various types of cancer cells [60].

Overall, low ROS levels appear to be beneficial for cancer cells as they can support their proliferative and invasive properties. However, beyond a certain threshold, ROS can become toxic to them. It seems that cancer cells can deploy an adaptive behavior to cope with different stages of ROS elevation (i.e., induce either pro-oxidant or antioxidant mechanisms) [53].

**The effect of reactive oxygen species on the components of store-operated calcium entry**

SOCE adaptation is a plausible mechanism of cellular adaptation to altered ROS levels. In particular, ROS modulate the function of Orai channels, thus regulating the calcium response, which is crucial for tumor growth. It has been demonstrated that endogenous and overexpressed Orai1 channels are inhibited by H_2_O_2_ with IC_50_ = 34 μM [61]. The same inhibitory effect was observed for Orai2 channels. In contrast, Orai3 channels were not inhibited by H_2_O_2_, indicating that Orai1 and Orai2 are sensitive to ROS, while Orai3 is not [61].

Cysteine residues are the primary targets for ROS in Orai1 and Orai2 [62]. In an Orai3 molecule, cysteine- 195 is replaced with glycine, which confers partial resistance to H_2_O_2_. Taking into account the differences in sensitivity to ROS among Orai1, Orai2, and Orai3, the ratio between these isoforms in the cell can be a factor that helps calcium signaling adapt to elevated ROS levels.

Similar processes are observed in immune cells. For example, an elevated Orai3/Orai1 ratio was revealed in monocytes, killing bacteria due to rapid H_2_O_2_ secretion; therefore, switching to the less ROS-sensitive Orai3 channels is an effective adaptation mechanism used by monocytes to withstand their own ROS production [63]. In primary human CD4^+^ T cells, naïve cells upregulate the Orai3/Orai1 expression ratio upon differentiation into effector cells residing within areas of the inflammation characterized by an elevated ROS concentration [61].

The ratio of expressed Orai proteins – and consequently the dependence of store-operated calcium entry on ROS – is altered not only in immune but also cancer cells (*Table 1*). Thus, reduced Orai3/Orai1 ratios have been observed in prostate cancer [64] and basal-like breast cancer cells [42]. However, elevated Orai3/Orai1 ratios have been reported in prostate cancer [65], as well as estrogen receptor-positive breast cancer [30, 42, 66] and non-basal-like breast cancer [42] as well. The differently directed changes in Orai channel expression in cancer cells are presumably driven by ROS, as well as other intrinsic and extrinsic factors within the tumor microenvironment.

As discussed above, STIM1 and STIM2 differ in terms of their sensitivity to the calcium level in the stores and ability to activate Orai channels. Furthermore, their sequences carry different oxidation- sensitive cysteine residues. STIM1 carries cysteine residues at positions 49 and 56, which can form a disulfide bond between each other in the presence of ROS [67]. Since cysteine 56 resides next to the Ca^2+^- binding domain of STIM1, it probably helps the protein to acquire a constitutively active form that activates SOCE, regardless of ER calcium levels [68]. Interestingly, the situation is diametrically opposite when these cysteine residues are oxidized by reactive nitrogen species. S-nitrosylation of cysteine residues C49 and C56 in STIM1 enhances the thermodynamic stability of its calcium-binding domain, thereby reducing its sensitivity to calcium and suppressing SOCE [69].

In contrast to STIM1, the STIM2 protein carries ten additional cysteine residues within its cytosolic domain. One of these STIM2-specific cysteine residues plays a crucial role in the context of the redox regulation of SOCE. Oxidation of cysteine C313 inhibits SOCE primarily by impeding STIM2 clustering, without affecting the STIM2–Orai1 interplay [70].

Therefore, both STIM proteins are sensitive to ROS-induced oxidation but via different mechanisms: STIM1 is modulated by ROS in the ER lumen, whereas STIM2 is inhibited by ROS in the cytosol.

**Adaptation of store-operated calcium entry to oxidative stress**

The mechanisms that alter the expression of SOCE proteins under oxidative stress have been identified. Simulation of 24-h oxidative stress in rat astroglioma cells led to a downregulated expression of STIM2, Orai1, and Orai3, and it also reduced the agonist-induced calcium response. However, the amplitude of SOCE and the degree of filling of the calcium stores remained virtually unchanged [71].

SOCE is highly susceptible to ROS. Sufficiently high ROS concentrations significantly, and nonselectively, affect the fundamental mechanisms maintaining cellular calcium homeostasis. In the case of adaptation to low ROS concentrations, the cell appears to have room for maneuver via the expression of different Orai channel isoforms.

## SUSCEPTIBILITY OF STORE-OPERATED CALCIUM ENTRY TO pH CHANGES

Compromised pH regulation is a shared characteristic of solid tumor cells. In most cases, these cells have an elevated intracellular pH (7.3–7.6 vs. normal pH 7.2) and reduced extracellular pH (6.8–7.0 vs. normal pH 7.4) compared to nontransformed cells [72]. The increased glycolytic activity in solid tumor cells leads to higher levels of lactate, protons, and carbonic acid in the extracellular environment, resulting in acidification of the tumor microenvironment [73]. Like hypoxia, acidification contributes to drug resistance from the tumor and immunosuppression within its microenvironment [74].

**The effect of pH changes on the components of store-operated calcium entry**

Fluctuations in pH levels significantly affect the functioning of numerous ion channels in the cell [75]. The influence of changes in extracellular and intracellular pH on the activity of Orai isoforms has been investigated rather well. Electrophysiological studies have demonstrated that changes in pH modulate both the endogenous SOCE and SOCE in HEK293 cells expressing exogenous STIM1/2 and Orai1/2/3 proteins. It turns out that extracellular acidification inhibits SOCE, while alkalinization potentiates it. Similarly, intracellular acidification reduces SOCE activation, whereas alkalinization accelerates the SOCE activation kinetics without altering the overall current amplitude [76]. Detailed studies demonstrated that the amplitude and kinetics of Orai1-mediated current are strongly dependent on the intracellular pH. The dependence of the current through Orai2 on intracellular pH manifests itself only as changes in amplitude. The Orai3 channel is totally independent of variations in intracellular pH [77]. It is most likely that intra- and extracellular pH regulate the activity of Orai channels through different mechanisms. Extracellular pH appears to modulate SOCE by affecting the Orai channel pore, while intracellular pH can affect aggregation and binding of STIM to Orai at several pH-sensitive sites. Thus, the H155F mutation in Orai noticeably reduces responsiveness to both acidic and alkaline intracellular pH values [78].

Since the amino acids E106, E190, and H115 are conserved in all three Orai isoforms, it is reasonable to assume that they act as common external sensors for acidic pH in all the Orai isoforms. Upon extracellular alkalinization, the amplitude of the current through all Orai channels increases (for Orai2, it rises to a larger extent compared to Orai1 and to a lesser extent compared to Orai3). It is possible that additional mechanisms governing the sensitivity of these channels to elevated pH levels exist [76, 78].

Interestingly, the STIM1-independent Orai1 mutant exhibits a reduced sensitivity to both intracellular alkalinization and acidification [77]. This fact can imply that, under conditions of changing intracellular pH, SOCE is regulated at the level of STIM proteins.

The effect of extracellular pH on other components of the calcium response remains insufficiently explored. The TRPC6 channel, which can be involved in SOCE, is known to be inhibited in acidic pH [79]. Intensification of research into the pH-dependent functioning of the proteins involved in calcium signaling can be anticipated in the coming years.

**Adaptation of store-operated calcium entry to changes in pH**

It still remains unclear whether changes in pH affect the expression of SOCE proteins in cancer cells; however, we know that pH has been shown to influence their clustering. Specialized clusters of SOCE proteins, known as calcium entry units (CEUs), are formed in muscle cells. The assembly of functional CEUs – including STIM and Orai proteins – is more intensive at elevated temperatures and reduced pH (i.e., upon intense muscle activity) [80]. Cluster assembly can perfectly be an additional mechanism of SOCE adaptation to a changing microenvironment of a higher order than the STIM–Orai interplay is. This mechanism enables the maintenance of the extracellular calcium influx essential for muscle function during transient acidification, thus preventing its reduction.

Hence, Orai channels, and possibly TRPC6, impede calcium overload of cancer cells under acidic tumor microenvironment conditions, which is caused by their sensitivity to extracellular acidification and reduced conductivity at low pH values. Acidification of the intracellular medium is accompanied by the involvement of additional mechanisms of SOCE regulation at the level of the interplay between the STIM and Orai proteins and the possibility to choose among the Orai isoforms.

## THE EFFECT OF HYPOXIA ON STORE-OPERATED CALCIUM ENTRY

Hypoxia is an essential factor within the tumor microenvironment closely related to cell proliferation, metabolism, angiogenesis, and the immune response. These processes frequently contribute to tumor progression and enhance its metastatic potential, including through the effect of hypoxia on the components of cellular calcium signaling [81].

**The effect of hypoxia on store-operated calcium entry components**

With respect to SOCE, hypoxic conditions contribute to the emptying of the ER calcium stores and elevation of the calcium concentration in the cytosol via two interrelated mechanisms: reduction of the cellular ATP level and production of low ROS levels.

Hypoxia may trigger STIM1 activation possibly by reducing the ATP level and pumping Ca^2+^ into the cellular store [82]. Hypoxia can also cause the depletion of intracellular calcium stores via the production of low ROS levels, rather than the reduction of the ATP level [83]. Emptying of calcium stores leads to the activation of SOCE, which is further weakened by hypoxia-induced acidification.

Hypoxia is known to cause rapid acidification of many cell types, including smooth muscle, cardiac, cancer, and neuronal cells [84, 85]. Under long-term hypoxia conditions, the cells of most tumor types become characterized by a high glycolysis rate and increased production of metabolic acids [86].

We have demonstrated the existence of a substantial inhibition of the calcium response under shortterm hypoxia in MCF-7 and BT-474 BC cells characterized by an elevated Orai3 level (*Table 1*) [36]. Contrariwise, an increased calcium response level under short-term hypoxia is observed in MDA-MB-231 and BT-20 cells characterized by a reduced Orai3 level [30, 36]. Hence, the resistance of cells to calcium overload under hypoxic conditions is dependent on the Orai3 level in the overall SOCE structure. On the other hand, hypoxia upregulates Orai3 expression [42]. Based on the aforementioned data, a conclusion can be drawn that expression of Orai proteins under long-term hypoxia in BC cells can be altered, with the Orai3 level increasing.

**Adaptation of store-operated calcium entry to hypoxia**

The Orai3 expression is upregulated under hypoxic conditions in many cancer cells: HCC1569, MDAMB- 231, MCF-7, and PMC42LA breast cancer cells, HT29 colon cancer cells, and Du145 prostate cancer cells. Furthermore, it has been demonstrated for the BC cell lines that changes in the expression levels of Orai3 are a response to long-term hypoxic conditions rather than the reason for the fluctuations in intracellular signaling [42].

The TRPC1 channel is another potential participant in the response to hypoxia in cancer cells [11]. TRPC1 expression is upregulated under hypoxic conditions in MDA-MB-231, MDA-MB-468, and HCC1569 breast cancer cell lines, but the expression levels of the homologous protein TRPC3 remain substantially unaltered [35]. Interestingly, suppression of TRPC1 expression in MDA-MB-231 and MDA-MB-468 cells increases the SOCE amplitude. This fact indirectly indicates that upregulated TRPC1 expression reduces the SOCE amplitude [35, 87]. In this case, similar to the Orai3 channel, the TRPC1 channel is involved in the cellular defense mechanism under hypoxic conditions.

Hence, the synergistic effect of several factors causing intracellular calcium imbalance, including acidification and ROS production, is witnessed under hypoxic conditions. The TRPC1 and Orai3 channels can confront these detrimental factors to a certain extent.

## CONCLUSIONS

**Fig. 4:**
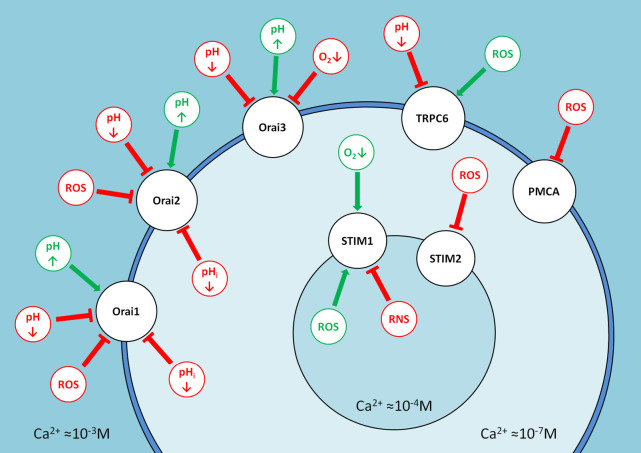
A schematic showing the effects of tumor microenvironment components on the store-operated calcium entry. The approximate concentrations of calcium in the cell, ER, and the extracellular matrix are specified. The major SOCE proteins are shown in black; the green and red colors denote the activating and inhibitory effects of the respective microenvironmental factors

Calcium plays an important role in oncogenesis processes due to its signaling function, as well as by ensuring the functioning of mitochondria [38, 39]. Various calcium signaling mechanisms are involved in the adaptation of cancer cells to the complex landscape of the tumor microenvironment (*Fig. 4*).

At an increasing ROS concentration, Orai3 ensures the functioning of SOCE, while inhibition of STIM2 prevents calcium overload in the cell. At acidic intercellular and intracellular pH values, conductivity of all the Orai channels suffers, except for Orai3, which is independent of the intracellular pH.

Along with the properties of SOCE proteins *per se*, their expression is also altered in response to stress conditions. Expression of the STIM2, Orai1, and Orai3 proteins is downregulated under oxidative stress; hypoxia upregulates the expression of the TRPC1 and Orai3 proteins. The reason behind the changes in the expression levels of channels is individual in each particular case (adaptation or a sequela of carcinogenesis); however, these alterations normalize the current tumor microenvironment rather than destabilize calcium signaling in the tumor. Therefore, the lower adaptative potential for cancer cells enhances the effectiveness of antitumor therapy and exerts an independent curative effect.

Many SOCE components are viewed as targets for antitumor therapy [26, 88]. There are certain challenges related to the narrow choice of selective SOCE modulators. Currently, the number of potential targets substantially surpasses the number of available modulators. Unfortunately, there are no selective modulators for most proteins involved in SOCE. For example, the Orai3 channel plays a crucial role in the adaptation of cancer cells to changes in microenvironmental pH, hypoxia, and an elevated ROS level. However, selective modulators for this channel remain to be identified. Meanwhile, both of the activators of this channel, which would lead to calcium overload in cancer cells characterized by upregulated Orai3 expression, and inhibitors that disrupt the overall calcium homeostasis in these cells, are of interest for therapeutic purposes. The existing selective SOCE inhibitors target the main calcium entry pathway through STIM1–Orai1 proteins, making these inhibitors highly toxic to the body [89]. They can be used only provided that the targeted delivery problem is solved; otherwise, the systemic harm from their administration outweighs the potential therapeutic benefit. The situation is somewhat better in the therapy of autoimmune diseases, where Auxora (also known as CM4620), a selective Orai1 inhibitor, exhibits a therapeutic effect, although this is accompanied by severe side effects [90]. Minor SOCE components, such as the proteins STIM2, TRPC1, and numerous adapter proteins (SARAF, α-SNAP, STIMATE, Junctate, IRE1, etc.), should be selected as targets to reduce the chances of systemic toxic effects on the body [23]. Previously, we have identified a modulator of the STIM2-dependent signaling pathway: the low-molecular-weight compound 4-MPTC that exerts an inhibitory effect on SOCE via the STIM2-dependent calcium entry pathway but does not suppress calcium entry through the STIM1-dependent pathway. The target of this compound is still to be identified [91].

A larger number of available selective modulators would enable fine-tuning of SOCE, enhance therapeutic versatility, reduce the adverse effects of therapy, and facilitate the transition toward personalized medicine.

## Abbreviations

SOC: store-operated channels
SOCE: store-operated calcium entry
SERCA: sarcoplasmic/ endoplasmic reticulum Ca2+-ATPase
PMCA: plasma membrane Ca2+-ATPase
ROS: reactive oxygen species
ER: endoplasmic reticulum
BC: breast cancer
